# Delayed Presentation of Anticonvulsant Hypersensitivity Syndrome Secondary to Lamotrigine

**DOI:** 10.7759/cureus.27399

**Published:** 2022-07-28

**Authors:** Rafeef O Jarwan, Mohsin Ali

**Affiliations:** 1 Preventive Medicine, King Abdulaziz University Hospital, Jeddah, SAU; 2 Family Medicine, King Saud Bin Abdulaziz University for Health Sciences, Jeddah, SAU

**Keywords:** maculopapular rash, anticonvulsant, epilepsy, fever, anticonvulsant hypersensitivity syndrome, lamotrigine

## Abstract

Anticonvulsant hypersensitivity syndrome (AHS) is a rare condition that needs to be seriously recognized and diagnosed. However, it is difficult to diagnose it since its clinical manifestation mimics other common infectious and neoplastic diseases. AHS manifests as skin rash that is preceded by fever accompanying internal organ involvement, with the liver being mostly affected. AHS is a condition that develops secondary to anticonvulsant exposure like phenytoin, phenobarbitone, carbamazepine, and lamotrigine. A defect in epoxide hydroxylase leading to the accumulation of toxic metabolites of aromatic anticonvulsant is hypothesized to play a role in developing AHS. This report presents a case of a 49-year-old epileptic Asian female, who complained of persistent high-grade fever followed by generalized maculopapular rash, high liver enzymes, and pancytopenia. The patient had unremarkable past history and systematic review. The patient went through various investigations to rule out infections and systematic diseases and all investigations were normal. After excluding every possible cause, the patient was diagnosed with AHS secondary to lamotrigine usage for one month.

## Introduction

Anticonvulsant hypersensitivity syndrome (AHS) is a rare and serious adverse reaction that can be potentially fatal if misdiagnosed, with a mortality rate of 10%-20% [1,2]. It is characterized by the manifestation of a triad including fever, rash, and internal organ involvement [1]. In the 1950s, AHS was described for the first time in history and it was estimated to occur in 1:1000 to 1:10,000 patients who were treated by anticonvulsants [2,3]. It has been commonly reported to occur with the use of phenytoin, phenobarbitone, carbamazepine, and lamotrigine [3]. AHS appears after a brief delay of 2-4 weeks of anticonvulsant usage, but it can appear up to 12 weeks of anticonvulsant exposure [3]. The exact mechanism of AHS is still not well understood [2]. In general, AHS is considered to be a type IV hypersensitivity reaction, which is a T-cell-mediated delayed reaction [2]. However, it is believed that AHS is related to genetic variations in the metabolism of anticonvulsants and it has been addressed that the abnormal metabolism problem is due to a defect in epoxide hydroxylase enzyme which is responsible for aromatic anticonvulsants' degradation leading to AHS symptoms [1,4]. The inheritance pattern of AHS is thought to be an autosomal-dominant pattern, and there is an increased chance of developing AHS in siblings of the affected patients [2]. It was found that the human leukocyte antigen HLA-A*3101 has an association with the cutaneous reaction secondary to carbamazepine exposure [2]. Also, there is an association of human herpesvirus 6 (HHV6), human herpesvirus 7 (HHV7), cytomegalovirus (CMV), and Epstein-Barr virus (EBV) with AHS [2]. In addition, since many reported cases of AHS are female patients, the hormonal effect is thought to be part of the AHS pathogenesis [2]. In a Spanish case report that was published in 2020, a case of a 25-year-old female epileptic patient who developed AHS after oxcarbazepine exposure for one month and 15 days was reported [4]. She presented with fever, diffuse pruritic morbilliform rash, facial angioedema, cervical adenopathy, axillary adenopathy, whitish vomiting, leukopenia, and high liver enzymes suggesting hepatitis [4]. Serology tests for Venereal Disease Research Laboratory (VDRL), human immunodeficiency virus (HIV), hepatitis B virus (HBV), and hepatitis C virus (HCV) were performed, and they came out negative [4]. Her condition became worse after increasing the dose of oxcarbazepine [4]. However, the patient's symptoms and abnormal lab results subsided after discontinuation of oxcarbazepine along with dexamethasone and diphenhydramine administration during hospitalization [4]. In another case report that was published in 2017, a 30-year-old American female patient with a history of bipolar disorder was presented to the hospital after 10 days of carbamazepine usage with AHS symptoms including generalized painful rash, nausea, vomiting, facial edema, malaise, mild thrombocytopenia, and a mild increase in liver enzymes [3]. Her condition was treated with the discontinuation of carbamazepine and intravenous methylprednisolone during hospitalization [3]. A case report that was published in 2017 presented a case of a 15-year-old female patient from the Gaza strip, Palestine [4]. She was found to have an intermittent high-grade fever, jaundice, diffuse maculopapular rash, axillary lymphadenopathy, inguinal lymphadenopathy, and moderate splenomegaly [4]. The patient was diagnosed with AHS after one month of phenytoin usage as seizure prophylaxis due to a previous head injury [4]. Additionally, exposure to sulfonamides, sulfones, allopurinol, and non-steroidal anti-inflammatory medication can cause a similar reaction to AHS, so this reaction has also been referred to as drug-induced hypersensitivity syndrome (DIHS) or drug-related rash with eosinophilia and systemic symptoms (DRESS) syndrome [3]. AHS is frequently undetected due to the failure of many physicians to recognize and treat AHS [1]. Since most AHS cases are underreported, the true incidence of this condition is still unknown [2]. Therefore, in this case report, we present an interesting case of a 49-year-old female patient who experienced AHS symptoms after approximately one month of lamotrigine exposure to treat her seizure disorder.

## Case presentation

A 49-year-old Asian female who was newly diagnosed with epilepsy on lamotrigine presented for the first time to the Employee Health Clinic with a complaint of a high-grade fever of 41°C for two days along with a sore throat. The high-grade fever was spiking despite taking antipyretics to treat the fever. The systematic review was unremarkable as there were no skin lesions, and neurological, respiratory, gastrointestinal, urinary tract, or musculoskeletal symptoms. The patient had undergone gastric bypass surgery five years ago. Also, she has been diagnosed with epilepsy four months ago and was well controlled on levetiracetam 750 mg BID. However, the patient could not tolerate levetiracetam, so it was switched to lamotrigine 50 mg BID. The patient started to use lamotrigine for one month before her first presentation to the clinic. The patient was a physician herself and had no close contact with any sick patients with similar symptoms. The travel history was negative and there were no reported insect or mosquito bites, contact with animals, ingestion of raw milk, or ingestion of raw meat. Additionally, the patient was not a smoker and did not use alcohol or illicit drugs. On examination, she was vitally stable apart from a fever of 41°C. She looked pale and dehydrated with injected throat but no exudates. The systemic examinations were normal. The lab showed mild raised C-reactive protein (CRP), normal procalcitonin, low white blood cell (WBC) count and lymphocytes 0.72 × 10^9^/L, normal neutrophils, and normal platelets. Lab results are shown in Table 1.

**Table 1:** Lab work on the day of the presentation

Exam	Result	Reference
C-reactive protein (CRP)	6.4 mg/L	0-5 mg/L
Procalcitonin	0.11 ug/L	<0.25 ug/L
White blood cells (WBC)	3.6 × 10^9^/L	5-19.5 × 10^9^/L
Lymphocytes	0.72 × 10^9^/L	2.1-13.8 × 10^9^/L
Neutrophils	2.13 × 10^9^/L	0.8-6.8 × 10^9^/L
Platelets	155 × 10^9^/L	150-450 × 10^9^/L

She was arranged to have the following tests: malaria smear, Brucella antibodies, PCR swab for COVID-19, influenza A and B, Middle East respiratory syndrome coronavirus (MERS-COV), parainfluenza viruses 1-2-3-4, respiratory syncytial virus, *Mycoplasma pneumoniae*, *Chlamydia pneumoniae*, *Bordetella pertussis*, coronaviruses 229E, HKU1, NL63, and OC43, *Human metapneumovirus*, adenovirus, human rhinovirus/enterovirus.

The next day, the patient was following up with the Employee Health Clinic. She reported a generalized maculopapular rash with few pustular lesions and all of the above investigations came back as undetected/normal. Given that all the above initial workup was negative with persistent fever and now rash, she was treated empirically with oral cefuroxime.

On day 3 of her presentation, the patient continued to have fever (39-40°C) and rash showed no improvement despite 48 hours of cefuroxime. Hence, she had labs repeated, which showed raised CRP, normal procalcitonin, high creatinine, low WBC, low lymphocyte, dropped platelets, low hemoglobin, and low sodium. Lab results are shown in Table 2.

**Table 2:** Day 3 lab work

Exam	Result	Reference
C-reactive protein (CRP)	0.7 mg/L	0-5 mg/L
Procalcitonin	0.19 ug/L	<0.25 ug/L
Creatinine	75 umol/L	28-47 umol/L
White blood cells (WBC)	3.4 × 10^9^/L	5-19.5 × 10^9^/L
Lymphocytes	0.66 × 10^9^/L	2.1-13.8 × 10^9^/L
Platelets	137 × 10^9^/L	150-450 × 10^9^/L
Hemoglobin	9.5 g/dL	10-14 g/dL
Sodium	134 mmol/L	135-144 mmol/L

Therefore, she was admitted and the examination findings were unremarkable except for small palpable tender bilateral occipital lymph nodes, dehydration signs, and pallor. Investigations to rule out EBV, CMV, TB, and dengue were ordered in addition to a chest x-ray (CXR), and discharged to be followed up after administration of IV acetaminophen and IV normal saline. The results of dengue, CMV, EBV serology, CXR, and urine culture were all reported as normal but she continued to spike fever of 400°C with generalized persistent rash but was otherwise hemodynamically well.

Five days after discharge, she had lab work repeated, which showed raised CRP, normal procalcitonin, high alanine aminotransferase (ALT), high aspartate aminotransferase (AST), low lymphocyte, low WBC, low neutrophils, low platelet, low hemoglobin, low sodium, and low chloride. Lab results are shown in Table 3.

**Table 3:** Lab work five days after discharge

Exam	Results	Reference
C-reactive protein (CRP)	18.7 mg/L	0-5 mg/L
Procalcitonin	0.19 ug/L	<0.25 ug/L
Alanine aminotransferase (ALT)	140 U/L	6-28 U/L
Aspartate aminotransferase (AST)	254 IU/L	5-34 IU/L
Lymphocytes	0.63 × 10^9^/L	2.1-13.8 × 10^9^/L
White blood cells (WBC)	2.3 × 10^9^/L	5-19.5 × 10^9^/L
Neutrophils	1.12 × 10^9^/L	0.8-6.8 × 10^9^/L
Platelets	140 × 10^9^/L	150-450 × 10^9^/L
Hemoglobin	9.6 g/dL	10-14 g/dL
Sodium	132 mmol/L	135-144 mmol/L
Chloride	99 mmol/L	101-11 mmol/L

The patient was readmitted as case of fever of unknown origin with generalized rash, pancytopenia, and abnormal liver profile. Differential diagnosis involved acute viral illness, drug reaction, malignancy, and autoimmune disease. Lab workup was repeated and the results showed low platelet, low hemoglobin, low mean corpuscular volume, low mean corpuscular hemoglobin, critically low WBC, low lymphocyte, low neutrophils, low eosinophil, low monocyte, high lactate dehydrogenase, high ALT, high AST, high CRP, high erythrocyte sedimentation rate, normal procalcitonin, and normal rheumatology markers. Lab results are shown in Table 4.

**Table 4:** Readmission lab work

Exam	Result	Reference
Platelets	144 × 10^9^/L	150-450 × 10^9^/L
Hemoglobin	8.9 g/dL	10-14 g/dL
Mean corpuscular volume (MCV)	70.2 fL	76-96 fL
Mean corpuscular hemoglobin (MCH)	23.9 pg	27-32 pg
White blood cells (WBC)	1.3 × 10^9^/L	5-19.5 × 10^9^/L
Lymphocytes	0.36 × 10^9^/L	2.1-13.8 × 10^9^/L
Neutrophils	0.69 × 10^9^/L	0.8-6.8 × 10^9^/L
Eosinophils	0.06 × 10^9^/L	0.1-1.6 × 10^9^/L
Monocytes	0.19 × 10^9^/L	0.2-0.8 × 10^9^/L
Lactate dehydrogenase (LDH)	362 U/L	100-217 U/L
Alanine aminotransferase (ALT)	140 U/L	6-28 U/L
Aspartate aminotransferase (AST)	254 IU/L	5-34 IU/L
C-reactive protein (CRP)	17.8 mg/L	0-5 mg/L
Erythrocyte sedimentation rate (ESR)	77 mm/h	0-20 mm/h
Procalcitonin	0.19 ug/L	<0.25 ug/L
Rheumatology markers	Normal	Normal

Then, additional labs were ordered to further investigate the pancytopenia to rule out viral infections including hepatitis A, B, C, parvovirus, and HIV, which all came back as negative for acute infections. Chest CT, and abdomen and pelvis CT were ordered and they showed unremarkable results.

Finally, lamotrigine-related drug reaction was confirmed by the triad of AHS including fever, rash, and high liver function test (LFT) with pancytopenia suggesting that internal organ involvement was present while all other differentials were ruled out. Thus, lamotrigine was tapered off to 25 mg twice per day with the addition of lacosamide 50 mg twice per day. Three days after discharge, lamotrigine was stopped. Finally, two weeks after discharge, the patient followed up in the outpatient clinic, the fever had subsided, the rash resolved, and lab normalized following stoppage of lamotrigine. Lab results are shown in Table 5.

**Table 5:** Lab work after lamotrigine termination

Exam	Results	Reference
Platelets	171 × 10^9^/L	150-450 × 10^9^/L
Hemoglobin	12.7 g/dL	10-14 g/dL
Mean corpuscular volume (MCV)	88 fL	76-96 fL
White blood cells (WBC)	5.9 × 10^9^/L	5-19.5 × 10^9^/L
Lymphocytes	1.76 × 10^9^/L	2.1-13.8 × 10^9^/L
Neutrophils	3.54 × 10^9^/L	0.8-6.8 × 10^9^/L
Eosinophils	0.10 × 10^9^/L	0.1-1.6 × 10^9^/L
Monocytes	0.52 × 10^9^/L	0.2-0.8 × 10^9^/L
Alanine aminotransferase (ALT)	10 U/L	6-28 U/L
Aspartate aminotransferase (AST)	13 IU/L	5-34 IU/L

## Discussion

AHS was first discovered in 1950 [2]. However, in recent days, there have been some efforts to reclassify AHS under the broader term “drug-induced hypersensitivity syndrome (DIHS)” [2]. AHS is characterized by fever, rash, and internal organ involvement [4]. The AHS criteria include fever, lymphadenopathy, maculopapular rash starting after three weeks of treatment, clinical symptoms that last for two weeks after the suspected drug was retired, abnormal liver profile, leukocytosis, atypical lymphocytes, or eosinophilia [1]. Frequently, the rash that is caused by AHS is preceded by fever and malaise [2]. Skin rash manifests in 90% of the patients with AHS, and the rash is mostly maculopapular eruption in character with subsequent desquamation during resolution [4]. The skin eruption can range from morbilliform eruption to toxic epidermal necrolysis [2]. It has been noticed that carbamazepine has a higher incidence of skin manifestation compared to both lamotrigine and phenytoin [4]. In contrast, topiramate and valproic acid have a rare incidence of skin reaction [4]. In AHS, internal organ involvement is the major cause of morbidity and mortality [1]. In 50%-60% of the cases, the liver is the mostly affected internal organ followed by Clara cell in the lungs and tubular cells in the kidney [4]. Hepatic involvement is presented with high levels of liver enzymes that can reach up to fulminant hepatic failure levels [4]. The hematological alteration that occurs with AHS includes leukocytosis >11 × 10^9^/L, at least 5% of atypical lymphocytes, or eosinophilia >1.5 × 10^9^/L [1].

What makes our case unique is that the 49-year-old female patient presented with symptoms of AHS accompanying pancytopenia and low levels of eosinophils. According to different reports that discussed this adverse event, leukopenia can come along with hyponatremia which was present in our case. Moreover, according to the literature, there are other possible problems that can occur with AHS, such as thrombocytopenia, hemolytic anemia, carditis, pneumonitis, conjunctivitis, and splenomegaly [4]. In the case we are presenting, thrombocytopenia was present in addition to microcytic anemia. The diagnosis of AHS was difficult because the clinical manifestation and lab results could mimic infectious and neoplastic disorders, so many laboratory investigations were ordered to rule out other common causes. Therefore, AHS is still a diagnosis of exclusion [1]. 

The treatment of AHS is mainly supportive [2]. The discontinuation of the offending agent is the primary management [2]. The administration of systematic corticosteroids is frequently used even though there have been no clinical trials conducted to prove systematic corticosteroids' efficacy [2]. The patient in our case was managed by suspension of lamotrigine after being gradually tapered off and followed up with both internal medicine and neurology. After a few weeks of follow-up, the patient's condition including fever and rash has improved along with her lab results. 

## Conclusions

AHS is a rare condition that can be fatal if not treated early. It is characterized by fever, rash, and involvement of internal organs. The cause of AHS is still poorly understood. However, it is mostly hypothesized to be due to a defect in the metabolism of aromatic anticonvulsants. The diagnosis of AHS is considered difficult due to its great similarity to other more common conditions. It is mainly managed by discontinuation of the offending drug. 
